# Biological characteristics and epidemiological insights into the zoonotic potential of *Colpodella* spp.: a scoping review

**DOI:** 10.1186/s40249-025-01361-1

**Published:** 2025-08-28

**Authors:** Yilin Zhao, Zhanxin Cao, Shizhu Li, Chunhong Du, Jiafu Jiang

**Affiliations:** 1 State Key Laboratory of Pathogen and Biosecurity, Academy of Military Medical Sciences, Beijing, 100071 China; 2 Yunnan Provincial Key Laboratory of Natural Epidemic Disease Prevention and Control Technology, Yunnan Institute of Endemic Diseases Control and Prevention, Dali, 671000 China; 3 School of Public Health, Kunming Medical University, Kunming, 650500 China; 4 Chinese Center for Disease Control and Prevention, National Key Laboratory of Intelligent Tracking and Forecasting for Infectious Diseases; Key Laboratory on Parasite and Vector Biology, National Institute of Parasitic DiseasesChinese Center for Tropical Diseases ResearchMinistry of HealthWHO Collaborating Centre for Tropical DiseasesNational Center for International Research on Tropical Diseases, Ministry of Science and Technology, Shanghai, 200025 China; 5 School of Global Health, Chinese Center for Tropical Diseases Research, Shanghai Jiao Tong University School of Medicine, Shanghai, 200025 China

**Keywords:** *Colpodella*, Apicomplexa, Biological characteristics, Diversity, Human infections risk, Zoonosis

## Abstract

**Background:**

*Colpodella* species are classified within the domain Eukaryota, specifically under the order Colpodellida, family Colpodellaceae, and genus *Colpodella*, which are close relative of the phylum Apicomplexa. These organisms are unicellular, predatory flagellates. In recent years, their frequent detection in animal tissues, vector insect samples, and particularly in human has garnered significant attention as an emerging zoonotic threat. This review is to scope the biological characteristics and epidemiological features of *Colpodella* species.

**Methods:**

The PubMed, Web of Science, and China National Knowledge Infrastructure databases were searched to identify studies in English or Chinese published before 4 February 2025. We searched for *Colpodella*-related nucleotide sequences released in the GenBank before 31 December, 2024. The literature and sequences were selected based on predefined inclusion criteria. We extracted the characteristics of *Colpodella* spp. from included articles and performed a phylogenetic analysis based on the included sequences.

**Results:**

Thirty-seven records and 83 sequences were included in the study, respectively. *Colpodella* spp. currently comprise only two formally named species, alongside at least 11 species uncultured or unnamed in GenBank. Their life cycle includes trophozoite and cyst stages, with nutrient acquisition mediated by myzocytosis. These organisms display structural and protein similarities in their apical complexes to apicomplexan protozoa, yet with distinct differences. They are currently found in a wide range of hosts, including humans, livestock, pets, wildlife and vectors, across multiple continents, including Europe, Asia, Africa, and the Americas. Phylogenetic analyses reveal that *Colpodella* spp. exhibit significant genetic diversity and can be divided into seven clades, each characterized by distinct host ranges and regional distributions, and three clades posed pathogenic potential and significant risk of human infection.

**Conclusions:**

This study systematically elucidates the broad host/vector range, genetic diversity and public health implications of *Colpodella* species based on comprehensive integrated genomic and epidemiological analyses. We recommend establishing active surveillance networks using clade-specific molecular markers for hosts and vectors in high-risk regions, incorporating *Colpodella* screening into routine diagnostics for fever cases of unknown origin with anaemia, prioritizing studies on isolation and cultivation, biological characteristics, and clade-specific in vitro invasion assays to elucidate the pathogenic mechanisms.

**Graphical Abstract:**

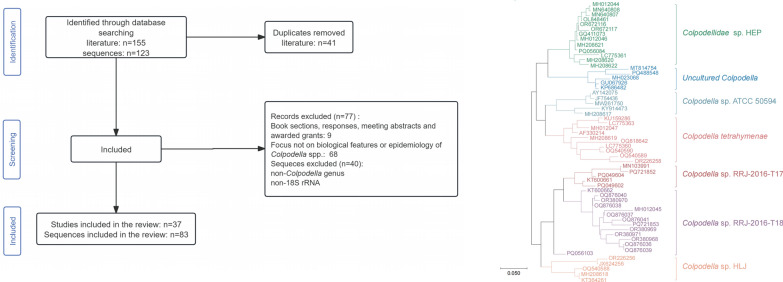

**Supplementary Information:**

The online version contains supplementary material available at 10.1186/s40249-025-01361-1.

## Background

Globally, approximately 70.0% of emerging infectious diseases originate from zoonotic pathogens, such as severe acute respiratory syndrome, Middle East respiratory syndrome, Ebola virus diseases, and Zika virus infection. Zoonoses significantly impact human livelihoods and well-being, posing a major threat to global economic development [1–3]. Driven by global integration, the expansion of human activities, and the emergence of pathogen mutations, there has been a resurgence of traditional zoonoses alongside a rising incidence of novel zoonotic diseases [4]. At least 250 zoonoses were listed as emerging and re-emerging zoonotic diseases during the last 70 years [5].

*Colpodella* spp. are a small group of free-living predatory protozoa, and feed on other protozoa, which have traditionally been considered non-pathogenic [6]. However, in recent years, there has been an increasing number of reports from different regions concerning *Colpodella* species among humans, wild and domestic animals, and vector insects such as ticks and flies, with growing evidence indicating their potential role as zoonotic pathogens. Especially, three human cases have been reported, causing different symptoms, with clinical manifestations and different strains of infection [7–9]. Animal infection studies have demonstrated that the symptomatic spectrum differs from that observed in human cases [10]. Genetic diversity plays a critical role in the adaptation and evolution dynamics of zoonotic pathogens, affecting their ability to infect diverse hosts and environments, and can cause different clinical and epidemiology character [11]. However, current knowledge regarding the genetic diversity and pathogenicity of *Colpodella* spp. remains limited. In addition, owing to limited clinical awareness of these protozoan infections, suboptimal diagnostic approaches, and the likelihood of substantial underdiagnosis, the reported cases likely represent only a fraction of the actual disease burden. These knowledge gaps present substantial obstacles to the accurate assessment of public health risks posed by *Colpodella* spp. as emerging zoonotic pathogens, highlighting the need for systematic studies in pathogen biology and epidemiology.

Therefore, this review focused on the biological characteristics and epidemiology of *Colpodella* spp. In order to gain deeper insights into the genetic diversity and evolutionary relationships of *Colpodella* spp., a comprehensive phylogenetic analysis of all available sequences of *Colpodella* spp. in GenBank will be conducted. Our objectives are to provide new insights for the proactive prevention of *Colpodella* spp. transmission and to highlight the necessity for further research into their zoonotic potential.

## Methods

This scoping review was conducted following the PRISMA Extension for Scoping Reviews (PRISMA-ScR) guidelines (Additional file 1) [12].

### Search strategy

A literature search was conducted in the database of PubMed, Web of Science and China National Knowledge Infrastructure (CNKI; https://www.cnki.net/), including publications in English or Chinese, with the search word “*Colpodella* [All Fields]”. Only articles published and indexed in the above databases up to 4 February 2025 were included and there was no beginning date cutoff. We used Endnote 20 (Clarivate, Philadelphia, USA) to manage these articles.

Nucleotide sequences related to *Colpodella* were retrieved from the GenBank (www.ncbi.nlm.nih.gov/genbank) with the search term “*Colpodella*” and “*Colpodellidae* sp. HEP”. The search results were restricted to sequences published before 31 December 2024.

### Inclusion criteria and exclusion criteria

#### Literature

Book sections, comments, responses, meeting abstracts and review articles were excluded. The inclusion criteria involved original, full-text articles, research on biological characteristics and/or epidemiology of *Colpodella* spp. We excluded articles that did not fit into the above themes.

#### Sequences

Sequences were screened based on their names and organism information, and only those explicitly labeled as belonging to the genus *Colpodella* were included. Exclusion criteria included sequences not belonging to the genus *Colpodella*, non-18S rRNA sequences.

### Data extraction and analysis

Two authors (YZ and ZC) independently searched through the literature and sequences. The two sets of papers and sequences were then compared. Disagreements were resolved by discussion or, if necessary, by including a third researcher (JJ) to make the final decision. Articles were categorized based on their content, including biological features and epidemiological studies (Additional file 2: Table S1). Relevant information was extracted and summarized. Sequence details, including name, accession number, features, and nucleotide sequence, were recorded for further analysis (Additional file 3: Table S3).

## Results

**Fig. 1:**
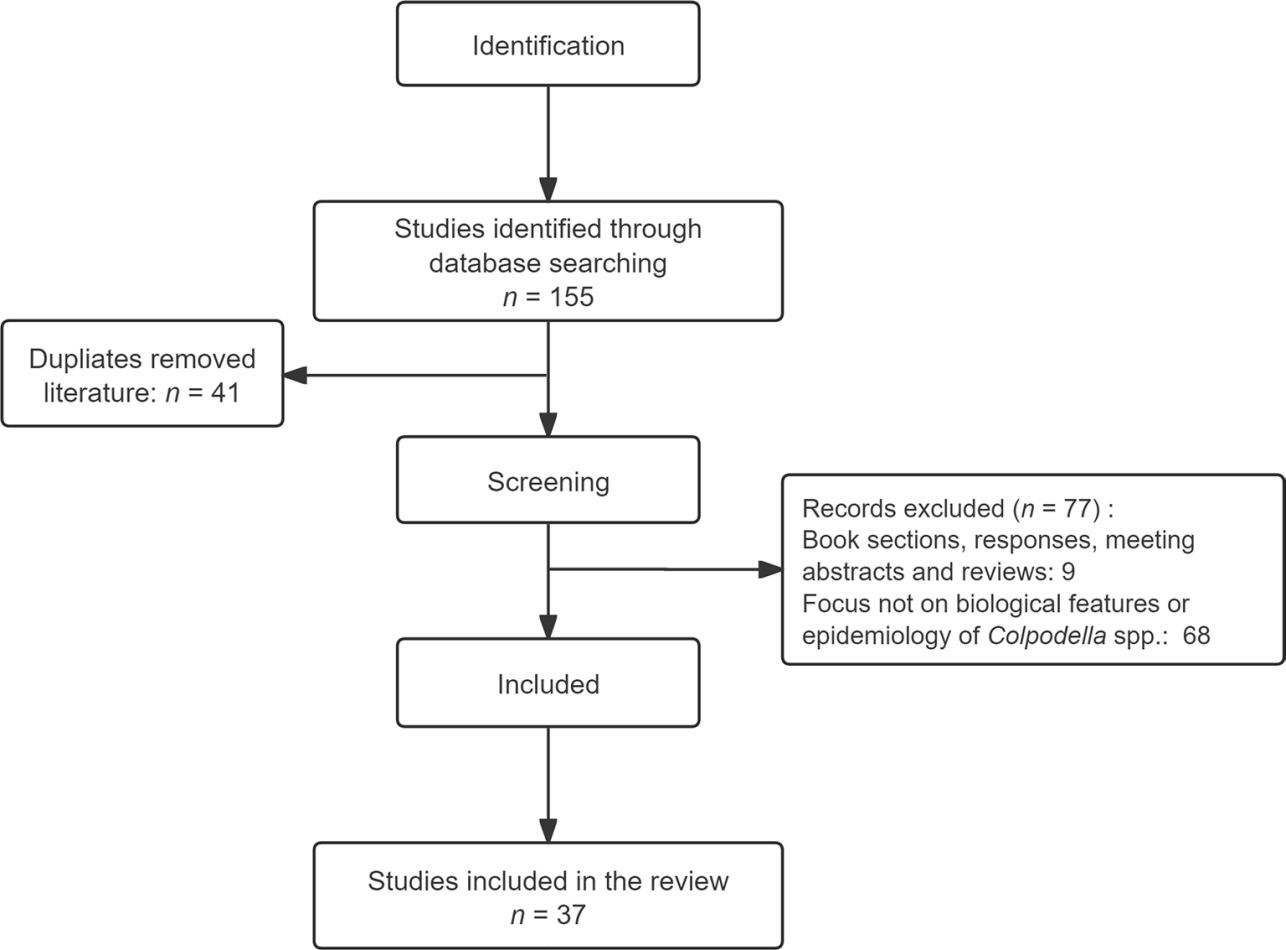
Flowchart of the literature screening process

**Fig. 2:**
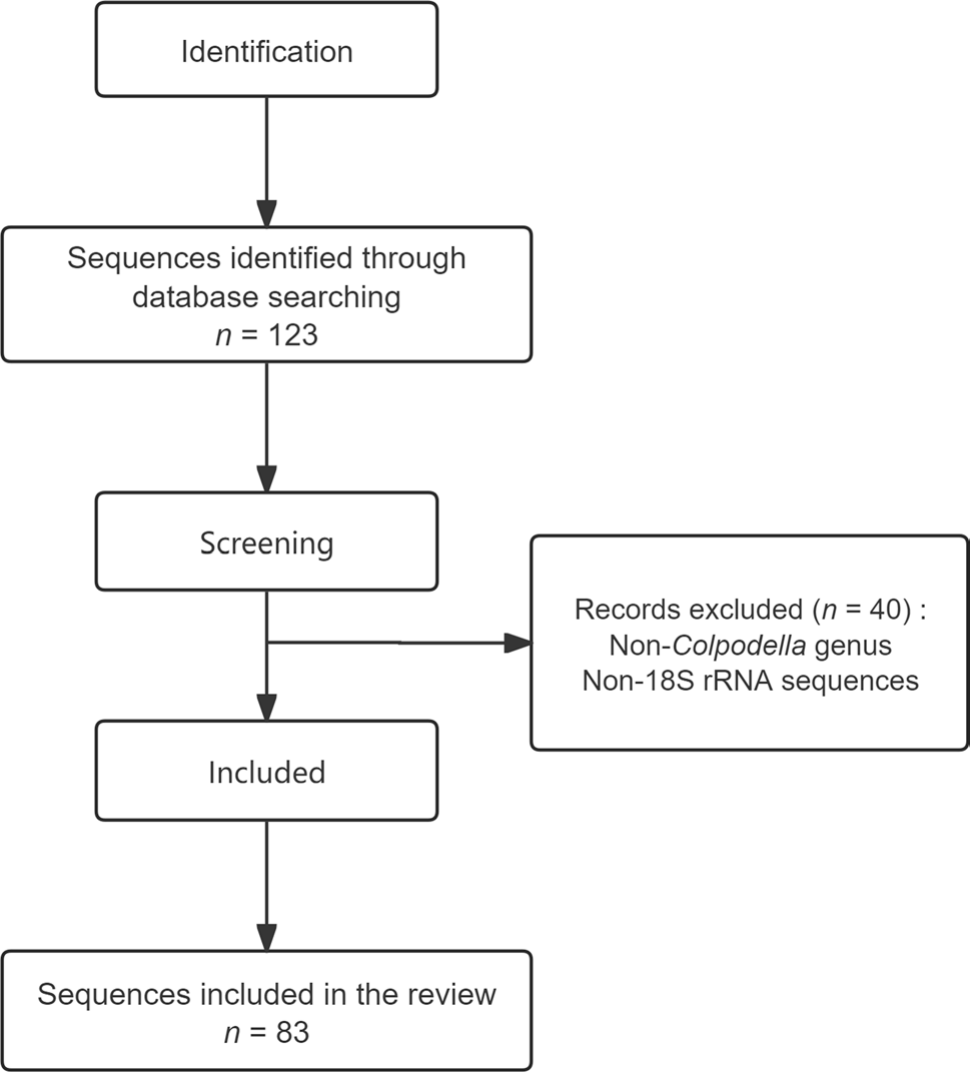
Flowchart of the sequences screening process

The systematic search identified 155 records, with 41 duplicate records removed. Following full-text review, 77 records were excluded according to the inclusion criteria. Ultimately, 37 articles met the inclusion criteria (Fig. 1). For genetic analysis, a total of 123 sequences were retrieved, and 83 sequences were included in the analysis based on the exclusion criteria (Fig. 2).

### Biological characteristics of *Colpodella* spp.

#### Taxonomic status and species of *Colpodella* spp.

*Colpodella* spp. are a small group of free-living predatory protozoans belonging to the the domain Eukaryota, order Colpodellida, family Colpodellaceae, and genus *Colpodella*. They primarily feed on protozoa and algae, have occasionally been identified in vertebrate hosts and arthropod vectors, and are phylogenetically closely related to apicomplexan protozoa, which comprise a diverse group of specialized intracellular parasites [6, 13, 14], including *Plasmodium*, *Toxoplasma*, and *Cryptosporidium*. Currently, *Colpodella* spp. include the formally described species *Colpodella tetrahymenae* and *Colpodella angusta*, as well as at least 11 unclassified and unnamed species in GenBank (Additional file 3: Table S3).

#### Morphological features of *Colpodella* spp.

Similar to other apicomplexan protozoa, *Colpodella* spp. possess micropores and apical complexes, which comprises rhoptries, micronemes, pseudo-conoids, polar rings and subpellicular microtubules [6, 15]. In contrast to the complete ring conoid of the apicomplexan protozoa, the pseudo-conoid of *Colpodella* spp. consists of an incomplete ring of microtubules [16]. The number of microtubules comprising the pseudo-conoids varies among *Colpodella* species. *C. angust* generally has 20–27 microtubules while *C. tetrahymenae* has only 4–5 [17]*.*

#### Life cycle of *Colpodella* spp.

The in vitro life cycle of *Colpodella* spp. comprises trophozoite stage and cyst stage. Traditional techniques (wet mounts and Giemsa-stained smears) enable observation of trophozoite movement and cyst detection [18, 19]. However, these methods can not distinguish between *Colpodella* sp. and prey cysts. Sam-Yellowe TY et al. [20] developed a novel trichrome staining technique that enabled detailed differentiation and observation of *Colpodella* sp. cyst stages.

The trophozoite stage is the active feeding phase of *Colpodella* spp. After constructing tubular connections to attach to prey, trophozoites suck its cytoplasm. After feeding, the anterior end of the trophozoite disintegrates, accompanied by the disappearance of flagella and organelles. The food vacuole, along with the remaining cytoplasm and nucleus, forms a pre-cyst, which subsequently differentiates into an early demilune cyst and initiates encystation [16, 21]. Most cysts of *Colpodella* spp. typically contain two or four juvenile trophozoites [6, 15, 16]. However, *Colpodella vorax* and *Colpodella* sp. ATCC 50594 exhibit cysts containing up to 10 juvenile trophozoites [16, 22]. Trophozoites emerge from cysts, appearing either as individual cells or as paired, resuming feeding activities to complete the life cycle. *Colpodella* sp. ATCC 50594 has a 36-h life cycle, with peak activity occurring between 20 and 28 h [21].

#### Mechanisms of nutrient uptake of *Colpodella* spp.

*Colpodella* spp. utilize myzocytosis for feeding. Myzocytosis, a primarily predatory trophic mode, which is based on penetration of the prey surface and sucking of its contents via specialised organelles morphologically similar to those used by the apicomplexan zoites for invasion [23]. Following attachment of the pseudo-conoids of *Colpodella* spp. trophozoites to the prey surface, the microtubules at the attachment site dilate, enabling the engulfment of the prey's cell membrane and cytoskeleton. This process establishes a specialized tubular connection between predator and prey, enabling directional transport of prey cytoplasm into the posterior food vacuole. At the end of feeding, *Colpodella* spp. detach from the prey surface and continue to search for new prey [17, 21, 22]. The most common predator–prey interaction involves the attachment of a single *Colpodella* individual to a single prey organism during feeding. In addition, instances of two to three *Colpodella* individuals simultaneously attaching to a single prey cell have been observed [17, 21]. Unlike pathogenic apicomplexan parasites, which form parasitophorous vacuol by invading host cells during feeding, *Colpodella* spp. form food vacuoles without invading their prey. Furthermore, micropores, which are present in all apicomplexan parasites, are hypothesized to function in endocytosis. In *Toxoplasma gondii*, the micropore has been identified as an essential organelle for nutrient uptake via endocytosis [24]. Except for *Colpodella* sp. ATCC 50594, endocytosis has not been reported in other *Colpodella* species [25]. Therefore, it remains unclear whether *Colpodella* spp. employ endocytosis as an additional feeding mechanism alongside myzocytosis. *Colpodella* species may utilise endocytosis to absorb and survive on nutrients in human, animal, and arthropod tissues. Alternatively, they may engage in contact-dependent interactions with cells, leading to cell and tissue destruction or invasion of human cells [26, 27].

#### The key role of apical complexes-related protein

Actin plays a critical role in the invasion of host cells by apicomplexan parasites, such as *T. gondii* and *Plasmodium falciparum* [28, 29]. Cytochalasin D blocks the action of actin in *T. gondii* [30]. Similarly, Cytochalasin D treatment of *Colpodella* sp. ATCC 50594 induces cytoskeletal deformation and disrupts the tubular tether during predation, indicating that actin is involved in tubular tether formation [31]. In addition, Apical complexes organelles use their related proteins to initiate host cell invasion and facilitate intracellular trophozoite survival [32]. *Colpodella* spp. also have an apical complex. Sam-Yellowe TY et al. demonstrated that antibodies to proteins involved in host cell invasion by apicomplexan parasites cross-reacted with apical proteins of *Colpodella* sp. ATCC 50594. Antibodies targeting the rhoptry protein RhopH3 of the *Plasmodium* species cross-react with the tubular connection structures formed between *Colpodella* sp. ATCC 50594 and its prey during the myzocytosis stage [33]. Similarly, antibodies against apicomplexan invasion-related proteins, such as inner membrane complex subunit 3 (IMC3), apical membrane antigen 1 (AMA-1), erythrocyte-binding antigen 175 (EBA-175), and Plasmepsin II, also showed cross-reactivity with this organism [21]. The cross reactivity of antibodies against apical complex proteins with *Colpodella* sp. ATCC 50594 proteins suggests that events of myzocytosis may have preceded events that led to zoite internalization within host cells in intracellular parasitism [21]. These findings suggest that the function of *Colpodella* spp. apical proteins may resemble that of apicomplexan parasites during host cell invasion, indicating that *Colpodella* spp. may interact with host cells through their proteins, potentially causing cellular damage and facilitating invasion.

Both feeding mechanism and the structure and function of their apical complex organelles of *Colpodella* spp. show significant similarities to those involved in host cell invasion by apicomplexan parasites. However, there is still a lack of research on the invasion mechanisms of *Colpodella* spp., even though Yuan et al. have observed *Colpodellidae* sp. HEP in human red blood cells [7].

### Epidemiological studies on *Colpodella* spp.

#### Human infections with *Colpodella* spp.

To date, *Colpodella* spp. have been identified in three patients (Table 1). The first case was reported in 2008 in Yunnan Province, China, who was a 57-year-old woman, presented with cough and malaise for 6 months and was found to have several hematological abnormalities. Microscopic examination for peripheral blood smear identified parasites resembling *Babesia* spp. Then she was treated with atovaquone and azithromycin. Retrospective PCR targeting the 18S rRNA gene and sequence analysis revealed she was infected with *Colpodella* sp. which had an 89.0% similarity with *C. tetrahymenae*, and was designated as *Colpodellidae* sp. HEP [7]. In 2018, our research team reported the second case of *Colpodella* infection, a 55-year-old female patient with a confirmed history of tick bite in Heilongjiang Province, China. Neurological examination revealed moderate nuchal rigidity. The initial clinical diagnosis was Lyme disease. Administration of doxycycline led to improvement in the patient’s condition. However, retrospective testing of blood and cerebrospinal fluid samples was negative for all known tick-borne pathogens. Unexpectedly, the recovered 18S rRNA sequence from her cerebrospinal fluid sample was closely related to *Colpodella* spp. (89.0–90.0% similarity) and was provisionally designated as *Colpodella* sp. HLJ [8]. Notably, in 2021, *Colpodella* sp. was identified in urine samples from a 70-year-old female patient with lung disease in Romania using wet mounts and Giemsa-stained smears. The patient presented with breathing difficulties and multiple chronic diseases, but exhibited no urinary pathology or symptoms before or during hospitalization. Treatment included ceftriaxone and metronidazole. Final urine sediment examinations prior to discharge confirmed the absence of the parasite [9].

**Table 1:** Description of human cases of *Colpodella* spp. infection

	Patient 1 [5]	Patient 2 [6]	Patient 3 [7]
Epidemiological characteristics			
Nation	China	China	Romania
Age (years)	57	55	70
Gender	Female	Female	Female
History of tick bite	N/A	√	N/A
Basic medical history	N/A	NA	COPD II, HF III, T2DM, Ob III
Clinical manifestations			
Fever	–	√	–
Headache	–	√	–
Dizziness	–	√	–
Malaise	√	–	–
Orthopnea	–	–	√
Cough	√	–	–
Gait disturbance	–	√	–
Nuchal rigidity	–	√	–
Laboratory findings			
Granulocyte proportion	–	↓	–
Hematocrit	↓	–	–
Hemoglobin	↓	–	–
Reticulocyte count	↑	–	–
Lactate dehydrogenase levels	↑	–	–
Serum AST or ALT or γ-GGT concentration	–	↑	–
Proteinuria	–	+	–
Immunoglobulin G antibody against Borrelia burgdorferi sensu lato	–	+	N/A
Smear	*Babesia* spp. like	N/A	Mobile parasitic trophozoites
Detection methods for *Colpodella* spp.	PCR	PCR	Wet mounts and Giemsa-stained smears
Treatment			
Antibiotics	√	√	√
Antiprotozoal drugs	√	/	√
Outcome	Recovery	Recovery	Recovery

*N/A* Not available,—Within normal limits, √ Yes, ↑ increase, ↓ decline, + positive, *COPD II* Chronic Obstructive Pulmonary Disease Grade 2, *HF III* Heart Failure III, *T2DM* Type 2 Diabetes Mellitus, *Ob III* Obesity Class III, *ALT* alanine aminotransferase, *AST* aspartate aminotransferase, *γ-GGT* γ-glutamyl transferase

These three cases suggest that *Colpodella* spp. may act as opportunistic pathogens in humans. However, as these cases were identified retrospectively, the target organs, pathogenicity, sources of infection, and transmission mechanisms remain poorly characterized. Although *Colpodella* sp. HLJ was initially associated with a tick bite in the patient, retrospective analysis of tick-derived sequences revealed significant divergence from the human isolate.

#### Genetic diversity of *Colpodella* spp.

Despite the limited number of reported human infections, the genetic diversity of *Colpodella* spp. among animals and vectors has been increasingly recognized through phylogenetic analyses. Understanding the genetic variation within this genus is crucial for elucidating its potential pathogenicity, host adaptation, and transmission dynamics.

**Fig. 3:**
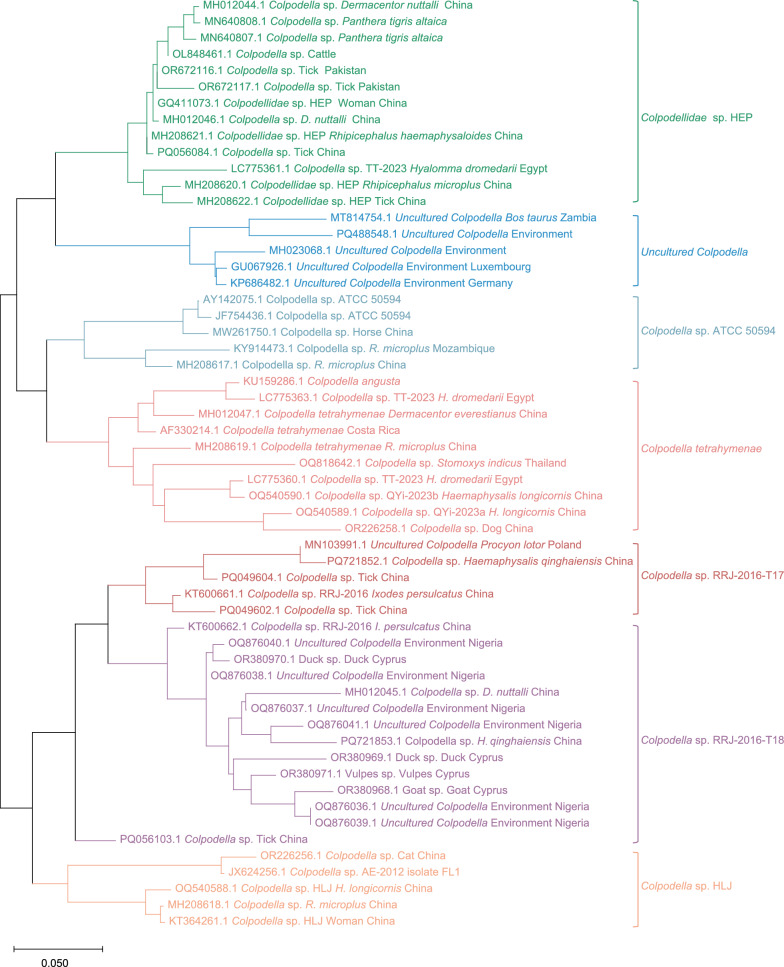
Phylogeny of *Colpodella* spp. sequences of 18S rRNA gene, using the Neighbor-Joining method with Bootstrap 1000 replicates. The phylogenetic tree was constructed using the MEGA 11.0 software. Sequences from the same institution, at the same time, and from the same source are considered to be from the same species if their similarity is > 99% [47], and only one is shown in the figure. For specific selection reasons, please refer to Table S3

A phylogenetic tree was constructed using all available 18S rRNA sequences of *Colpodella* spp. retrieved from GenBank (Fig. 3). The phylogenetic tree was divided into seven distinct clades based on sequence similarity (> 87.0%) between the most closely related sequences in adjacent branches. Species classification was conducted using a similarity threshold of 95% provisionally, with each of the seven clades containing multiple *Colpodella* species (Additional File 2: Table S2). Representative *Colpodella* species were provisionally named for each clade, including *Colpodellidae* sp. HEP., *Uncultured Colpodella*, *Colpodella* sp. ATCC 50594, *Colpodella tetrahymenae*, *Colpodella* sp. RRJ-2016-T17, *Colpodella* sp. RRJ-2016-T18, *Colpodella* sp. HLJ. To date, only two *Colpodella* strains, *Colpodellidae* sp. HEP (accession numbers: GQ411073) and *Colpodella* sp. HLJ (accession numbers: KT364261), have been associated with human infections, while nearly 15 strains are linked to animals and vectors. The hosts of *Colpodella* spp. in clades of *Colpodellidae* sp. HEP and *Colpodella* sp. HLJ include wildlife, livestock, ticks, pets and humans, suggesting a high potential for an animal-vector-human transmission cycle. Although the clades of *Colpodella* sp. ATCC 50594, *Colpodella* sp. RRJ-2016-T17, *Colpodella* sp. RRJ-2016-T18 and *Colpodella tetrahymenae* have not been found to be directly associated with human infections, the host ranges of these species encompass wildlife, pets, livestock, and vector insects such as ticks and flies. This wide host distribution suggests a potential risk of transmission to humans. In addition, the clade of *Uncultured Colpodella* is currently only associated with livestock, with additional environmental detections..

#### Geographical distribution of *Colpodella* spp.

Over the past two decades, *Colpodella* spp. have been identified to be widely distributed across multiple continents-Asia, Europe, the Americas, and Africa, inhabiting diverse geographical regions and ecological environments (Additional file 4: Table S4). *Colpodella* species currently exhibit a broad geographic distribution, with all six clades (excluding the *Uncultured Colpodella* clade) reported across Asia. The *Colpodella* sp. HLJ clade currently exhibits a relatively limited geographical distribution pattern, mainly distributed in Heilongjiang Province, Yunnan Province, Shandong Province and Guizhou Province in China. Notably, the *Colpodellidae* sp. HEP clade has been detected in both Asia and Africa, while clades of *Colpodella* sp. RRJ-2016-T17 and *Colpodella* sp. RRJ-2016-T18 show transcontinental distributions, occurring in Europe—with the latter additionally present in Africa. Most remarkably, the clades containing *Colpodella* sp. ATCC 50594 and *C. tetrahymenae* demonstrate the widest distribution range, spanning Africa, Asia, and the Americas. The widespread distribution of *Colpodella* spp. suggests not only a broad ecological niche but also a strong environmental adaptability [14, 34, 35]. These characteristics together promote their potential for cross-regional spread.

#### Risk of human infection with *Colpodella* spp.

First, the widespread distribution of *Colpodella* spp. further increase their likelihood of infecting humans. *Colpodella* spp. were frequently detected not only in hosts and vectors, but also in a wide range of environmental samples. For example, *Colpodella* sp. ATCC 50594 was originally isolated from brown woodland soil samples in Gambrill State Park, Maryland, United States [14]. In addition, *Colpodella* spp. have also been identified in specific reef environments, including coral tissue, mucus, and associated seawater sediments [35]. Similarly, *Colpodella* spp. were also detected in water samples collected from small ditches near a tiger park in China [34]. These findings suggest that *Colpodella* spp. may also be transmitted through environmental media such as soil and water bodies.

Second, the extensive prevalence of *Colpodella* spp. within zoo animals (e.g., tigers), domestic animals (e.g., horses), pets (e.g., dogs and cats) and wildlife (e.g., foxes and birds) in intimate contact with humans substantially escalates the likelihood of human infection. In China, *Colpodella* spp. have been detected in fecal samples from different felines (*Panthera tigris, Panthera leo*, *Panthera onca, Lynx lynx*) from Harbin Zoo, Heilongjiang Province [36], blood from horses in Ordos, Inner Mongolia [37], pet cats and dogs in Guizhou Province [38]. A South China tiger (*Panthera tigris amoyensis*) in Fujian Province was bitten by ticks and exhibited clinical symptoms such as severe jaundice throughout the body, enlarged liver and spleen, and bleeding in the kidneys and lymph nodes [34]. The sequence of *Colpodella* sp. detected in the blood of the South China tiger showed 90.1% similarity to *Colpodellidae* sp. HEP and 90.4% similarity to *Colpodella* sp. HLJ. Given the close contact between humans and these animals, the risk of infection with *Colpodella* spp. is significantly increased.

Humans can become infected with *Colpodella* spp. not only through daily contact with domestic animals and pets but also through increasing interactions with wildlife. In Cyprus, *Colpodella* spp. were identified in fecal samples from Cyprus red foxes (*Vulpes vulpes indutus*), Eurasian coots (*Fulica atra*) and ducks (*Anas* sp.) [39].

Third, blood-sucking ticks and flies, as important vectors, will play a significant role in the transmission of *Colpodella* spp. among people. The patient, from Heilongjiang Province, China, had a history of tick bite. During field investigations in the woodlands surrounding the patient's living area in Heilongjiang Province, 474 host-seeking adult *Ixodes persulcatus* ticks were screened, among which two were identified as positive for *Colpodella* spp. [8]. In China, different *Colpodella* spp. were also detected in other tick species: ticks (*Dermacentor nuttalli*, *Dermacentor abaensis*, *Haemaphysalis qinghaiensis*) across nine counties in Qinghai Province [40], goat-attached *Haemaphysalis longicornis* ticks in Shandong Province [41], *Amblyomma javanense* ticks on *Malayan pangolins* intercepted by customs in Guangdong Province [42] and ticks (*Haemaphysalis* spp., *Dermacentor* spp., *Rhipicephalus* spp.) around a tiger park, in Fujian Province [34], further suggesting that ticks may act as their vectors. In addition to findings in China, *Colpodella* spp. were also identified in *Hyalomma dromedarii* ticks infesting camels in southern Egypt [43], as well as in *Rhipicephalus microplus* ticks that feed on cattle in Mozambique [44]. Moreover, this microorganism has been detected in *Stomoxys indicus* flies that are associated with horses in Thailand [45]. Strong associations between *Colpodella* spp. and multiple tick species and other arthropods suggest that they may serve as key vectors for their transmission and spread.

Through a thorough review of literature on the distribution of *Colpodella* spp.in the environment, animals and vectors (Table 2), we demonstrated the wide geographical distribution and host range of *Colpodella* spp. Potential transmission routes for human infection with *Colpodella* spp. may include vector-borne transmission and indirect contact transmission. Being bitten by infected vectors or coming into contact with contaminated objects can lead to infection *Colpodella* spp. In addition, *Colpodella* spp. have also been detected in animal feces in current studies [36, 39], indicating that fecal–oral transmission may also exist. These findings highlight the complexity of their transmission routes and pose a potential threat to public health that cannot be ignored.

**Table 2:** Carriage records of *Colpodella* spp. in animals and arthropod vectors

Year of publication/isolation	Location	Isolation source	Sample	References
1993	America	Brown woodland soil	Environmental samples	[14]
2017	Mozambique	Cattle	*R. microplus*	[44]
2018	Coral reefs	Coral tissue, mucus, and associated seawater and sediments	Environmental samples	[35]
2018	Heilongjiang Province, China	N/A	*I. persulcatus*	[8]
2018	Qinghai Province, China	N/A	*D. nuttalli*, *D. abaensis*, *H. qinghaiensis*	[40]
2020	Poland	Raccoons	Ear fragments	[48]
2020	Zambia	Cattle	Blood	[49]
2022	Harbin Zoo, China	Felines	Feces	[36]
2022	Guangxi Province, China	Horses	Blood	[50]
2022	Cambodian	Dogs	Blood	[51]
2022	Inner Mongolia Province, China	Horses	Blood	[37]
2022	Heilongjiang Province, China	N/A	*Haemaphysalis concinna*	[52]
2022	Fujian Province, China	South China tiger	*Haemaphysalis flava*	[34]
		N/A	*H. flava*, *H. longicornis*, *Haemaphysalis hystricis*, *Haemaphysalis bispinosa*, *Dermacentor andersoni, Dermacentor atrosignatus*, *Dermacentor taiwanensis*, *Rhipicephalus duttoni*	
		Water samples	Environmental samples	
2023	Guizhou Province, China	Pet cats	Blood	[38]
		Pet dogs	Blood	
2023	America	Pet cat	Blood	[53]
2023	Thailand	N/A	*S. indicus*	[45]
2024	Zhejiang Province, China	Dog	*Rhipicephalus sanguineus*	[54]
2024	Shandong Province, China	Dogs	*H. longicornis*	[41]
		Goats	*H. longicornis*	
2024	Southern Egypt	Camels	*H. dromedarii*	[43]
2024	Italy	Cattle	*Rhipicephalus bursa*	[55]
2024	Guangdong Province, China	Pangolins	*A. javanense*	[42]
2025	Cyprus	Goats	Feces	[39]
		Foxes	Feces	
		Birds	Feces	

*N/A* Not applicable

## Discussion

In the present study, we conducted a systematic literature review focusing on the biological characteristics of *Colpodella* spp., as well as their epidemiological distribution and insights into the zoonotic potential factors, especially a comprehensive analysis of all available sequences of *Colpodella* spp. in GenBank from diverse sources. The finding could provide reference for proactive prevention and control diseases that may be caused by *Colpodella* spp.

### Pathogenic potential of *Colpodella* spp. and risk of human infection

In this study, we firstly explored the biological characteristics of *Colpodella* spp. Although there is currently no direct evidence of their pathogenicity, we have found similarities with apicomplexan protozoa in terms of structures and proteins associated with invasion of host cells.

Then, we revealed the genetic diversity of *Colpodella* spp. targeting all 18S rRNA sequences, which was divided into seven distinct clades. Notably, two clades, *Colpodellidae* sp. HEP and *Colpodella* sp. HLJ, are closely related to human infections, while the others are also primarily linked to infections in animals and vectors. Although the hosts of other clades are not directly related to humans, they include vectors, pets, livestock, and wildlife, and are extremely diverse. The broad geographic distribution and diverse host range of *Colpodella* spp. suggest these species may be transmitted through a wide variety of pathways, resulting in a complex animal-vector-human transmission cycle. Notably, distinct clades of *Colpodella* species may induce varying clinical manifestations. *Colpodellidae* sp. HEP (accession numbers: GQ411073) mainly caused coughing, general discomfort and haemolytic anaemia (elevated lactate dehydrogenase and increased reticulocyte count), while *Colpodella* sp. HLJ (accession numbers: KT364261) caused fever accompanied by neurological symptoms. This difference may reflect the specificity of different branches in the host cell invasion pathway. Recently animal infection studies have further expanded our understanding of pathogenic mechanisms and zoonotic potential of *Colpodella* spp. Wu et al. identified *Colpodella* RRJ-2016, *Colpodella* sp. HLJ, and *C. tetrahymenae* in cases of diarrhoea in yak and Tibetan sheep/goats, suggesting that certain clades may be enterophilic [10]. Soliman et al. detected *Colpodella* sp. (accession number: PQ380976) from sick camels with fever and diarrhoea, which showed 95.97% sequence similarity with *Colpodella* RRJ-2016-T18 (accession number: KT600662) [46]. Although the fatal South China tiger (*Panthera tigris amoyensis*) cases reported by Chiu et al. could not be phylogenetically classified due to unavailable sequence data, the observed multiorgan failure underscores the significant pathogenic potential of *Colpodella* spp. [34]. These findings indicate that distinct phylogenetic clades of *Colpodella* spp. may be associated with specific clinical manifestations, suggesting clade-dependent pathogenicity.

### Current research gaps

Currently, only *Colpodellidae* sp. HEP and *Colpodella* sp. HLJ have been definitively confirmed to infect humans. The limited number of reported human cases of *Colpodella* spp. infection hinders the identification of consistent clinical presentations and effective treatment strategies. The specific pathological mechanism of *Colpodella* spp. is currently unclear. Furthermore, research on the morphological structure, life cycle, and molecular mechanisms of *Colpodella* spp. capable of infecting humans and animals remains limited. Key aspects, including infection pathways, transmission dynamics, and disease manifestations, are not yet fully understood. Additionally, the structural and protein similarities between *Colpodella* spp. and apicomplexan protozoa raise a critical question: Do *Colpodella* spp. share pathogenic mechanisms, clinical diagnostic approaches, and therapeutic strategies with apicomplexan protozoa? Furthermore, in the *Colpodella* spp. infection studies included in this research, approximately 91.7% (22/24) were detected solely through polymerase chain reaction (PCR) identification of the 18S rRNA gene, lacking morphological evidence. This has limited clinical diagnosis and research on *Colpodella* infections. These uncertainties complicate the assessment of *Colpodella* spp. as zoonotic pathogens and underscore the need for more rigorous and in-depth research.

### Proactive prevention and control strategies

To proactively prevent and control *Colpodella* spp. infections, comprehensive measures need to be taken. Especially when the pathogenicity of *Colpodella* spp. have not yet been confirmed, morphological identification is often time-consuming and laborious. PCR-based monitoring can be implemented for early detection and intervention. In clinical practice, it is necessary to strengthen symptom monitoring, especially for unexplained fever symptoms, and to combine morphological identification with molecular biological testing for diagnosis. Treatment plans should be flexibly adjusted based on the patient's specific symptoms and the severity of their condition.

Furthermore, in high-risk areas where *Colpodella* species known to infect humans are present, implement evidence-based monitoring protocols targeting animal hosts and environmental vectors.

### Strengths and limitations

There are some notable strengths in the current study. To the best of our knowledge, this is the first study to explore the risk of human infection with *Colpodella* spp. from an epidemiological perspective, examining geographic distribution, host range, and genetic evolution. Phylogenetic analysis identified two clades that associated with human infectious. We found that *Colpodella* spp. have an increased likelihood of forming a animals-vector-human transmission cycle due to their broad host range, wide geographic distribution, and high genetic diversity. Despite the strengths of this study, there are still some limitations. First, studies published in languages other than English or Chinese were not included on account of the limits of languages, which may introduce language bias. Additionally, when constructing the phylogenetic tree, we found that some short sequences could not be effectively aligned with other 18S rRNA gene sequences. Therefore, in cases where sequences of the same species had already been included, we chose to exclude them to ensure the accuracy and reliability of the phylogenetic analysis. The phylogenetic analysis in this study was based on the 18S rRNA gene, as it has the most complete database and cross-group comparability in *Colpodella* spp. and related protozoa. Other genes such as ITS and cox1 were not used, mainly because of the extreme scarcity of available sequences (GenBank coverage < 10%) and the lack of standardized amplification protocols suitable for *Colpodella* spp. In the future, genomic data will need to be obtained through separate cultivation, and resolution will be improved through multi-gene joint analysis.

## Conclusions

This study systematically elucidates the broad host/vector range, genetic diversity and public health implications of *Colpodella* species based on comprehensive integrated genomic and epidemiological analyses. Especially, whole-genome phylogenetic analysis identified seven distinct clades, with three clades associated with human infections. We recommend establishing active surveillance networks using clade-specific molecular markers for hosts and vectors in high-risk regions, incorporating *Colpodella* screening into routine diagnostics for fever cases of unknown origin with anaemia, prioritizing studies on isolation and cultivation, biological characteristics, and clade-specific in vitro invasion assays to elucidate the pathogenic mechanisms.

## Supplementary Information

Additional file 1. PRISMA-ScR Checklist.

Additional file 2. Table S1: Included studies on the biological characteristics and epidemiology of *Colpodella* spp. Table S2: Genetic distance range within each evolutionary clade.

Additional file 3. Table S3: Sequences information of *Colpodella* spp. that meet the criteria in GenBank (before 31 December 2024).

Additional file 4. Table S4: Global distribution of *Colpodella* spp. in GenBank.

## Data Availability

Sequences data for *Colpodella* spp. in this paper were obtained from GenBank (www.ncbi.nlm.nih.gov/genbank).

## Abbreviations

PRISMA-ScR: Preferred Reporting Items for Systematic Reviews and Meta-Analyses Extension for Scoping Reviews
CNKI: China National Knowledge Infrastructure
IMC3: Inner membrane complex 3
AMA-1: Apical membrane antigen 1
EBA-175: Erythrocyte-binding antigen 175
PCR: Polymerase chain reaction
