# Supplementary material for: Biological characteristics and epidemiological insights into the zoonotic potential of Colpodella spp.: a scoping review
Source: Infect Dis Poverty. 2025 Aug 28;14:91. doi: 10.1186/s40249-025-01361-1 (PMC12392618; doi:10.1186/s40249-025-01361-1)
Supplement: Supplementary file 2 — Additional file 2. Table S1: Included studies on the biological characteristics and epidemiology of Colpodella spp. Table S2: Genetic distance range within each evolutionary clade. [file 40249_2025_1361_MOESM2_ESM.pdf]

Table S1 Included studies on the biological characteristics and epidemiology of *Colpodella* spp.

| Classification | | Reference |
|---|---|---|
| Biological characteristics of *Colpodella* spp. | Taxonomic status and species of *Colpodella* spp. | Simpson et al. [10]; Kuvardina et al. [11]; Siddall et al. [12] |
| | Morphological features of *Colpodella* spp. | Simpson et al. [10]; Cavalier-Smith et al.[13]; Brugerolle [14]; Mylnikov et al. [15] |
| | Life cycle of *Colpodella* spp. | Simpson et al. [10]; Cavalier-Smith et al. [13]; Brugerolle [14]; Sam-Yellowe et al. [16,18,20]; Yadavalli et al. [17]; Getty et al. [19] |
| | Mechanisms of nutrient uptake and host invasion in *Colpodella* spp. | Mylnikov et al. [15]; Getty et al. [19]; Sam-Yellowe et al. [20,23,29,31] |
| Epidemiology of *Colpodella* spp. | | Yuan et al. [5]; Jiang et al. [6]; Neculicioiu et al. [7]; Siddall et al. [12]; Chiu et al. [32]; Mathur et al. [33]; Hussain et al. [34]; Xu et al. [35, 51]; Wu et al. [36]; Hasapis et al. [37]; Han et al. [38]; Qi et al. [39]; Li et al. [40]; Soliman et al. [41]; Matsimbe et al. [42]; Phetkarl et al. [43] Solarz et al. [47]; Squarre et al. [48]; Zhou et al. [49]; Huggins et al. [50]; Wheatley et al. [52]; Zhu et al. [53]; Jimale et al. [54] |

Table S2 Genetic distance range within each evolutionary clade

| Clade name | Similarity (%) | |
|---|---|---|
| | Minimum | Maximum |
| *Colpodellidae* sp. HEP | 89.1 | 99.8 |
| *Uncultured Colpodella* | 84.1 | 99.0 |
| *Colpodella* sp. ATCC 50594 | 81.6 | 99.2 |
| *Colpodella_tetrahymenae* | 80.4 | 98.5 |
| *Colpodella* sp. RRJ-2016-T17 | 89.6 | 98.9 |
| *Colpodella* sp. RRJ-2016-T18 | 84.7 | 100 |
| *Colpodella* sp. HLJ | 80.4 | 99.7 |
